# Optimizing Metal Sites in Hierarchical USY for Selective Hydrocracking of Naphthalene to BTX

**DOI:** 10.3390/molecules30194023

**Published:** 2025-10-09

**Authors:** Kunyi Zheng, Mingjia Liu, Haidong Li, Xiu Chen, Xilong Wang

**Affiliations:** 1 School of Chemistry and Chemical Engineering, Linyi University, Linyi 276000, China; zhengkunyi2026@163.com; 2 State Key Laboratory of Heavy Oil Processing, China University of Petroleum, Beijing 102249, China; 13691005988@163.com

**Keywords:** hierarchical porous USY zeolite, bimetallic catalysts, naphthalene, selective hydrocracking, BTX

## Abstract

Metal components (CoMo, NiMo, NiW) supported on hierarchical porous USY zeolite (HPY) were systematically optimized for selective naphthalene hydrocracking to BTX (benzene, toluene, xylene). The hierarchical porosity enhanced mass transport and accessibility to active metal sites, improving reaction selectivity and efficiency. Supported metal sulfides served as hydrogenation sites, crucial for aromatic ring activation and coke suppression. By optimizing the synergy between hydrogenation and cracking functions, the optimized Ni_1_W/HPY catalyst achieved complete naphthalene conversion with a BTX yield of 92.5%. The spatial distribution of WO_3_ crystallites facilitated functional separation, promoting selective conversion. These findings underscore the importance of metal–acid balance and pore architecture in designing efficient hydrocracking catalysts.

## 1. Introduction

The strategic upgrading of light cycle oil (LCO)—a low-value refinery stream rich in polycyclic aromatic hydrocarbons (PAHs) like naphthalene—into high-purity benzene, toluene, and xylene (BTX) addresses dual industrial priorities: mitigating environmental impacts from transportation fuels and meeting growing demand for petrochemical feedstocks [1,2,3,4]. This conversion represents a crucial pathway for enhancing the value of refinery streams while supporting the transition toward more sustainable chemical production. Selective hydrocracking of PAHs to BTX relies critically on bifunctional catalysts that synergize metal-catalyzed hydrogenation with acid-driven cracking [5,6,7,8,9,10]. The intricate balance between these two functions determines both catalytic activity and product distribution, making the design of such catalysts particularly challenging.

Hierarchical pore engineering in USY zeolites has emerged as a powerful strategy to enhance reactant diffusion and accessibility to active sites [11,12,13]. By introducing mesopores while preserving the intrinsic microporous structure, these modified zeolites overcome diffusion limitations that often plague conventional microporous catalysts when processing bulky molecules like PAHs. However, while pore architecture optimization has received significant attention, the strategic design of metal components (e.g., CoMo, NiMo, NiW) remains equally critical yet less explored. The choice of metal component fundamentally dictates catalytic efficiency and product distribution, as it influences hydrogenation activity, sulfidation degree, and metal–acid balance [14,15,16,17,18,19,20,21,22].

Recent studies have highlighted the importance of optimizing metal dispersion and acidity to minimize over-cracking and coke formation [23,24,25,26,27,28]. Shin et al. discovered that when loading metals with varying tetralin hydrogenation (HYD) activities on H-Beta, hydrocracking (HYC) catalysts containing moderate-HYD-activity metals (CoMo-S, NiMo-S) exhibited higher BTX through-yield than those with high-HYD-activity metals (Ni, NiSn, NiW-S) or monofunctional H-Beta catalysts [10]. Peng et al. [29] evaluated three bifunctional catalysts for LCO upgrading: Ni-W/HY-Al_2_O_3_, Ni-W/Hβ-Al_2_O_3_, and Ni-Mo/HY-Al_2_O_3_. The Ni-Mo/HY-Al_2_O_3_ system showed the highest hydrocracking ability attributed to its balanced acidity and uniform metal dispersion. These discrepancies highlight the need to correlate metal speciation (dispersion, sulfidation state) with acidity profiles in hierarchical frameworks. The spatial distribution of metal sites relative to acid centers appears particularly important for controlling reaction pathways. Despite these advances, the relationship between metal speciation, pore architecture, and catalytic performance in hierarchical USY supports remains insufficiently understood. Many studies focus either on metal function or acid function separately, without considering their spatial relationship within hierarchical pore systems.

This work aims to systematically address this knowledge gap by optimizing metal sites (CoMo, NiMo, NiW) on hierarchical porous USY zeolite (HPY) for selective naphthalene hydrocracking. We specifically investigate how metal type and loading influence the spatial distribution of active phases and how this distribution affects the critical metal–acid balance required for high BTX selectivity. Through a suite of characterization techniques (XRD, N_2_ physisorption, TEM, XPS, H_2_-TPR, NH_3_-TPD, Py-FTIR) and catalytic evaluation, we elucidate the structure–activity relationships and identify the optimal metal composition for maximizing BTX yield.

## 2. Results and Discussion

### 2.1. Characterization of Catalysts

#### 2.1.1. Structure Properties and Pore Structure

**Figure 1:**
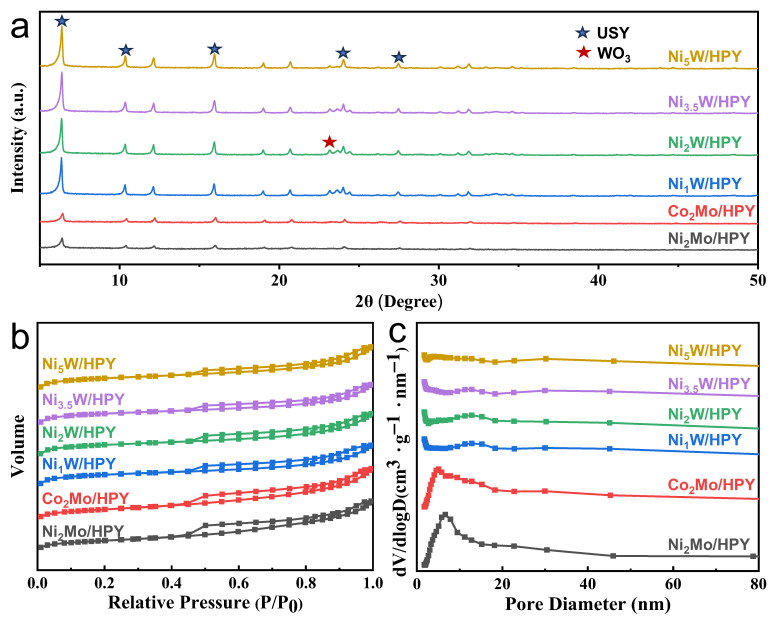
(**a**) Wide-angle XRD patterns, (**b**) N_2_ physisorption isotherms, (**c**) pore width distribution of supports.

XRD patterns (Figure 1a) confirm the successful synthesis of the core–shell structures. Characterization of the synthesized catalysts provided deep insights into their structural and chemical properties, directly correlating with their performance in naphthalene hydrocracking. XRD analysis revealed distinct metal dispersion patterns. Ni_2_Mo/HPY and Co_2_Mo/HPY catalysts exhibited only the characteristic peaks of the USY structure, indicating highly dispersed Mo species. In contrast, Ni_x_W/HPY catalysts displayed peaks at 2θ = 23.1° corresponding to crystalline WO_3_, signifying W agglomeration [30]. Crucially, the intensity of the WO_3_ peak was highest for Ni_1_W/HPY and progressively decreased with increasing Ni loading (Ni_2_W to Ni_5_W), suggesting that Ni promotes W dispersion on the HPY support at higher loadings.

Nitrogen physisorption further elucidated pore structure evolution (Figure 1b). While all catalysts exhibited type IV isotherms with H4 hysteresis loops (indicating particle-aggregation mesopores), pore size distributions revealed critical distinctions (Figure 1c). Ni_2_Mo/HPY and Co_2_Mo/HPY displayed broad mesopore distributions centered at ~7 nm, with moderate S_BET_ (258–277 m^2^/g), low S_mi_^c^ (170–194 m^2^/g), low V_mi_^c^ (0.07–0.08 cm^3^/g), and higher V_mes_ (0.17–0.20 cm^3^/g), confirming that their metal loading enhanced mesoporosity at the expense of microporosity [16].

In stark contrast, the Ni_x_W/HPY series exhibited significantly higher S_BET_ (536–548 m^2^/g), S_mi_^c^ (427–439 m^2^/g), and V_mi_^c^ (0.20–0.21 cm^3^/g), alongside lower V_mes_ (0.11–0.12 cm^3^/g). Critically, these catalysts maintained >90% micropore retention. This superior microporosity preservation stems from the unique interaction between larger WO_3_ crystallites inherent to NiW loading and the hierarchical pore architecture of the USY support. Specifically, the larger WO_3_ particles preferentially occupy mesopores and external surfaces, thereby minimizing blockage of the crucial micropore network essential for acid-catalyzed cracking. This selective deposition exposes more active sites accessible to nitrogen adsorption, increasing both measured microporous and mesoporous surface areas.

The preserved microporosity and strategic localization of metal species create a spatial segregation of functions: NiW active metals predominantly load onto the mesopores and external surfaces (regions with inherently lower acid site density), while the intact micropores house the strong Brønsted acidic cracking centers. This architectural optimization is essential for enforcing the desired sequential reaction pathway—initial hydrogenation of aromatic rings like naphthalene on the metal sites located in more open mesopores [31], followed by selective cracking of the hydrogenated intermediates (e.g., tetralin, decalin) within the sterically constrained micropores [32]. This bifunctional synergy, facilitated by the hierarchical USY structure interacting effectively with large W crystallites, enhances target product selectivity (Table 1).

#### 2.1.2. Microstructure and Morphology

**Figure 2:**
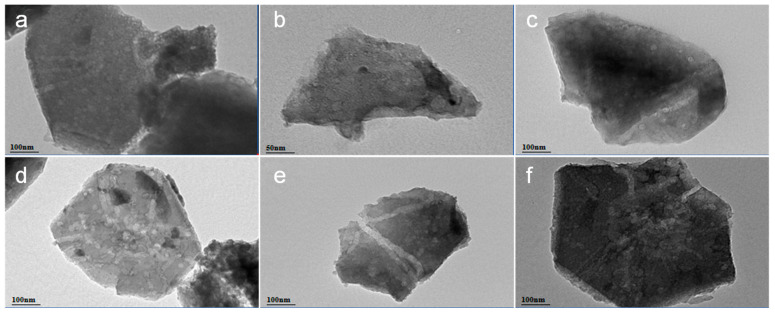
TEM image of A_x_B/HPY series catalysts: (**a**) Ni_2_Mo/HPY (**b**) Co_2_Mo/HPY; (**c**) Ni_1_W/HPY; (**d**) Ni_2_W/HPY; (**e**) Ni_3.5_W/HPY; (**f**) Ni_5_W/HPY.

TEM images confirmed the preservation of the intrinsic zeolite pore structure and morphology (slit-shaped pores, secondary pores) across all catalysts. No large metal agglomerates were readily observable, consistent with XRD indicating dispersion for NiMo/CoMo and suggesting that despite detectable WO_3_ XRD peaks, W is partially dispersed, with agglomerates likely being small or electron-transparent (Figure 2).

#### 2.1.3. Surface Acidity Properties of A_x_B/HPY Catalysts

**Figure 3:**
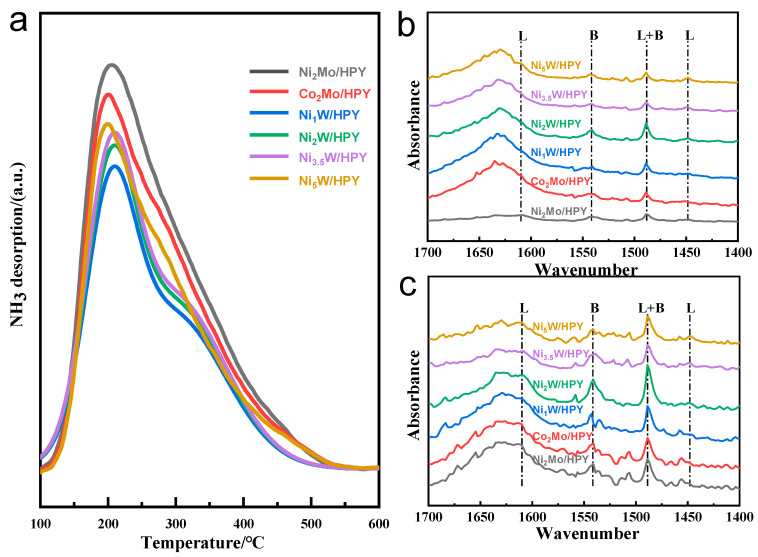
(**a**) NH3-TPD spectra of catalysts, (**b**) Py-IR spectra after desorbing at 200 °C, (**c**) Py-IR spectra after desorbing at 350 °C.

The acid properties of the Ni-loaded catalysts were characterized by NH_3_-TPD (Figure 3a) and pyridine-adsorbed FTIR (Py-IR, Figure 3b), with quantitative acid type distribution and strength summarized in Table 2. Total acidity and medium/strong acidity were determined by Py-FTIR at 200 °C and 350 °C, respectively, while Py-IR analysis identified Lewis acid sites (L) via peaks at 1450 cm^−1^ and 1610 cm^−1^, Brønsted acid sites (B) at 1540 cm^−1^, and overlapping Brønsted–Lewis sites (B + L) at 1490 cm^−1^ [33,34,35]. The Ni_2_Mo/HPY series exhibited the highest total acidity and strong acid density, followed by Co_2_Mo/HPY, while the Ni_x_W/HPY catalysts with varying Ni/W ratios consistently showed the lowest acid concentrations. Critically, excessive strong acidity (particularly Brønsted type) promotes undesired overcracking through β-scission and hydrogen transfer reactions, generating light hydrocarbons (C_1_–C_4_) at the expense of BTX yield due to accelerated coking and reduced aromatic selectivity.

#### 2.1.4. Metal–Support Interaction of A_x_B/HPY Catalysts

**Figure 4:**
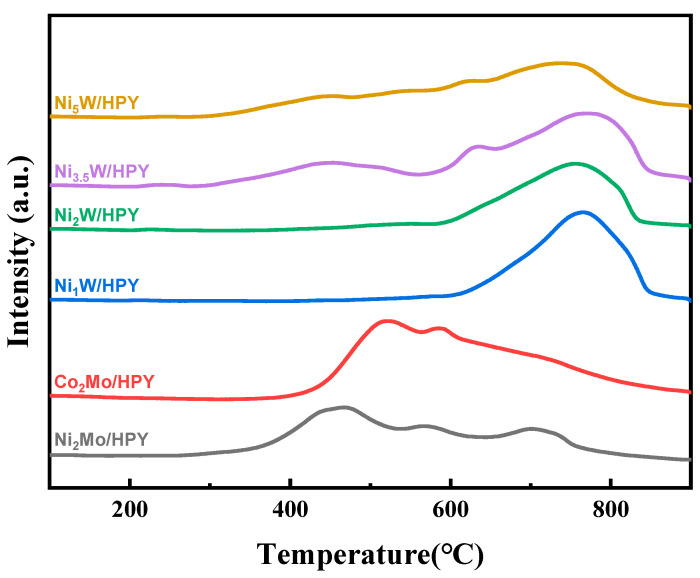
H_2_-TPR characterization of A_x_B/HPY series catalysts.

H_2_-TPR provided insights into reducibility and metal–support interactions (Figure 4). Ni_2_Mo/HPY and Co_2_Mo/HPY showed two main reduction peaks: a low-temperature peak (~420–520 °C) assigned to the reduction of octahedral Mo^6+^ species to Mo^4+^, and a high-temperature peak (~700–750 °C) for the further reduction of Mo^4+^ to Mo^0^ [36,37,38]. The Co-promoted catalyst exhibited a more intense low-temperature peak shifted slightly higher, indicating enhanced Mo dispersion/sulfidation and a stronger interaction with the support, consistent with the XPS findings. For Ni_x_W/HPY, Ni_1_W/HPY and Ni_2_W/HPY displayed only a high-temperature reduction peak (~750 °C) corresponding to WO_3_ reduction, with no distinct reduction peak for NiO (~400 °C), confirming excellent Ni dispersion at these low loadings [39,40]. Catalysts with Ni loading ≥ 3.5 wt% (Ni_3_._5_W/HPY, Ni_5_W/HPY) exhibited a clear NiO reduction peak around 400 °C, signifying Ni agglomeration. Furthermore, the WO_3_ reduction peak shifted to lower temperatures with increasing Ni loading (from ~750 °C for Ni_1_W to lower temperatures for Ni_5_W), signifying a weakening of the W–support interaction induced by Ni addition.

#### 2.1.5. Chemical State Analysis of Active Phase

**Figure 5:**
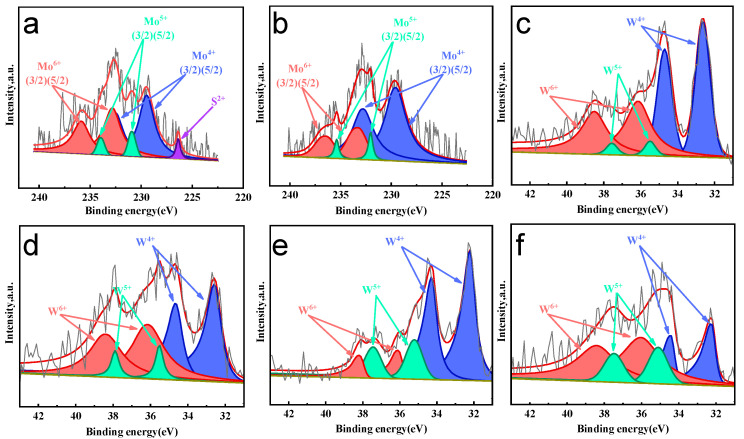
Mo(W) XPS spectra of sulfided A_x_B/HPY series catalysts: (**a**) Ni_2_Mo/HPY, (**b**) Co_2_Mo/HPY, (**c**) Ni_1_W/HPY, (**d**) Ni_2_W/HPY, (**e**) Ni_3.5_W/HPY. (**f**) Ni_5_W/HPY.

XPS analysis of sulfided catalysts focused on the chemical state of the active metals (Figure 5). Mo(W) XPS peak data of molybdenum (tungsten) species in sulfided AxB/HPY series catalysts summarized in Table 3. Co_2_Mo/HPY exhibited the highest sulfided Mo^4+^ content (72.4%), significantly exceeding that of Ni_2_Mo/HPY (48.8%), indicating that Co promotes Mo sulfidation on the HPY support. XPS analysis of sulfided Ni_x_W/HPY catalysts revealed tungsten’s complex speciation, where W 4f spectra deconvoluted into three distinct states: sulfided W^4+^ (WS_2_-like phase; W 4f5/2: 35.9 eV and W 4f3/2: 37.8 eV), partially sulfided W^5+^ (oxo-sulfide intermediate; W 4f5/2: 36.5 eV and W 4f3/2: 38.3 eV), and oxidized W^6+^ (WO_3_-like; W 4f5/2: 38.7 eV and W 4f3/2: 39.0 eV) [41,42,43]. Critically, Ni loading exerted a non-monotonic influence: Low Ni loading (Ni_1_W/HPY) maximized W^4+^ content (70.25%) by weakening strong metal–support interactions (MSIs) through Ni-O-W bond formation, which enhanced reducibility.

### 2.2. Catalytic Performance in Naphthalene Hydrocracking

**Figure 6:**
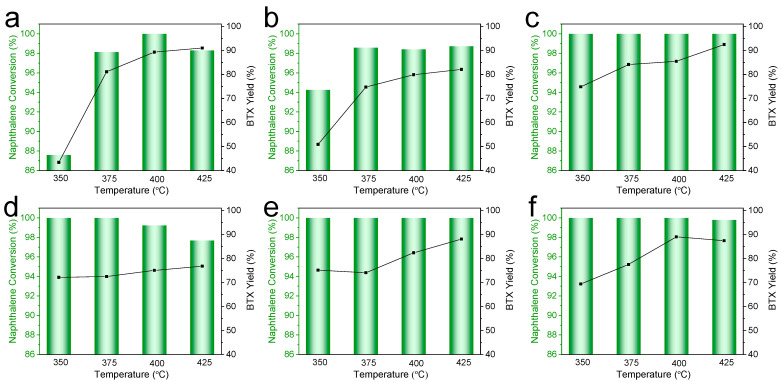
Conversion and BTX yield of naphthalene hydrocracking at different reaction temperatures over (**a**) Ni_2_Mo/HPY, (**b**) Co_2_Mo/HPY, (**c**) Ni_1_W/HPY, (**d**) Ni_2_W/HPY, (**e**) Ni_3.5_W/HPY, and (**f**) Ni_5_W/HPY.

Catalytic evaluation in naphthalene hydrocracking demonstrated clear performance differences (Figure 6). Ni_1_W/HPY delivered exceptional performance, achieving 100% naphthalene conversion across the entire temperature range tested (350–425 °C). Its BTX yield increased with temperature, reaching a maximum of 92.5% at 425 °C. In comparison, Ni_2_Mo/HPY showed lower naphthalene conversion at the lower temperatures and achieved a maximum BTX yield of 91% at 425 °C. Co_2_Mo/HPY attained high naphthalene conversion but yielded significantly less BTX than Ni_2_Mo/HPY. The other Ni_x_W/HPY catalysts (Ni_2_W to Ni_5_W) maintained high naphthalene conversion (>95% across temperatures) but yielded less BTX than the optimal Ni_1_W/HPY. Performance deteriorated significantly for Ni_5_W/HPY. For all catalysts except Ni_1_W/HPY (already at 100%), naphthalene conversion generally increased with temperature. BTX yield typically peaked at an intermediate temperature (e.g., 425 °C for Ni_1_W/HPY and Ni_2_Mo/HPY), beyond which overcracking likely reduced yield.

The outstanding performance of Ni_1_W/HPY arises from a unique synergy between metal properties and the HPY support, enabling optimal metal–acid balance and spatial separation. The formation of large WO_3_ crystallites, evident from XRD, and their preferential location within mesopores and on the external surface of the HPY particle, confirmed by the exceptionally high micropore surface area and volume (S_mi_^c^ = 427 m^2^/g, V_mi_^c^ = 0.20 cm^3^/g) in N_2_ physisorption, physically separates the primary hydrogenation sites (sulfided W species in meso/external zones) from the primary cracking sites (strong Brønsted acid sites within the preserved micropores, Py-FTIR: ~196.5 µmol/g strong B-acid). This spatial separation enforces an efficient “hydrogenate then crack” pathway: naphthalene is rapidly hydrogenated to tetralin on the accessible W sites; tetralin diffuses into the micropores where strong Brønsted acids catalyze selective ring-opening and dealkylation to BTX. This separation minimizes undesired deep hydrogenation of tetralin to decalin on metal sites and prevents rapid poisoning of the vital acid sites by metal species or large polyaromatic intermediates. The low Ni loading (1 wt%) is critical: it minimizes Ni agglomeration (H_2_-TPR: absence of NiO peak) and strong metal–support interaction (H_2_-TPR: high WO_3_ reduction temperature), allowing for a high degree of W sulfidation (XPS: 70.25% W^4+^), thereby maximizing the concentration of the active hydrogenation phase (WS_2_). While higher Ni loadings (x > 1) in Ni_x_W/HPY improve W dispersion (reduced WO_3_ XRD peak intensity), they simultaneously strengthen the W-support interaction (H_2_-TPR peak shift to lower temperature), hinder complete sulfidation (reduced W^4+^ XPS content), and eventually lead to Ni agglomeration (H_2_-TPR NiO peak), collectively reducing hydrogenation activity and overall catalyst efficiency. Although Co_2_Mo/HPY demonstrated superior Mo sulfidation (XPS: 72.4% Mo^4+^ vs. Ni_2_Mo/HPY: 48.8%), likely due to the formation of the highly active “CoMoS” phase, its lower total and strong Brønsted acidity (NH_3_-TPD, Py-FTIR) compared to Ni_2_Mo/HPY resulted in inferior cracking performance and consequently lower BTX yield, indicating Ni_2_Mo/HPY offered a better balance under the specific conditions of this study.

## 3. Experimental Section

### 3.1. Materials

Naphthalene, cobalt nitrate hexahydrate, ammonium molybdate tetrahydrate, ammonium metatungstate hydrate and nickel nitrate hexahydrate were purchased from Sinopharm Chemical Reagent Co., Ltd. (Beijing, China). USY zeolite was purchased from Shanghai Macklin Biochemical Co., Ltd. (Shanghai, China). hydrochloric acid was purchased from Beijing Chemical Plant (Beijing, China).

### 3.2. HPY Support Synthesis

USY zeolite was treated with 0.2 mol/L HCl solution at a solid-to-liquid volume ratio of 1:10. The mixture was stirred in a round-bottom flask at 80 °C for 4 h. The solid was then filtered, washed repeatedly with ultrapure water until neutral pH, dried, and calcined to obtain the HPY support.

### 3.3. Catalyst Synthesis (Sequential Incipient Wetness Impregnation)

In this study, the synthesized catalysts are denoted as AxB/HPY, where ‘A’ represents the promoter metal (Ni or Co), ‘x’ denotes the mass fraction (wt%) of the promoter metal oxide (e.g., NiO or CoO), ‘B’ represents the primary active metal (Mo or W), and HPY stands for the hierarchical porous USY support.

Active metals were loaded onto the HPY support. Total metal oxide loading was fixed at 18.5 wt%. Ni_2_Mo/HPY (2 wt% NiO/16.5 wt% MoO_3_), & Co_2_Mo/HPY(2 wt% CoO/16.5 wt% MoO_3_): Ammonium heptamolybdate tetrahydrate ((NH_4_)_6_Mo_7_O_24_·4H_2_O) was first dissolved in deionized water, ultrasonically dispersed for 15 min, impregnated onto HPY, followed by ultrasonication, drying, and calcination. After cooling, nickel nitrate hexahydrate (Ni(NO_3_)_2_·6H_2_O) or cobalt nitrate hexahydrate (Co(NO_3_)_2_·6H_2_O) was dissolved, impregnated, ultrasonicated, dried, and calcined to obtain Ni_1_Mo/HPY or Co_2_Mo/HPY.

Ni_x_W/HPY (where x denotes the mass fraction of NiO) Series (x = 1, 2, 3.5, 5): Ammonium metatungstate hydrate ((NH_4_)_6_H_2_W_12_O_40_) was first dissolved in deionized water, ultrasonically dispersed for 15 min, impregnated onto HPY, followed by ultrasonication, drying, and calcination. After cooling, nickel nitrate hexahydrate (Ni(NO_3_)_2_·6H_2_O) was dissolved, impregnated, ultrasonicated, dried, and calcined to obtain Ni_x_W/HPY.

### 3.4. Catalyst Characterization

The structure and physicochemical properties of the supports and catalysts were investigated using various characterization techniques. These included X-ray diffraction (XRD), transmission electron microscopy (TEM), ammonia temperature-programmed desorption (NH_3_-TPD), N_2_ adsorption–desorption isotherms, H_2_ temperature-programmed reduction (H_2_-TPR), and X-ray photoelectron spectroscopy (XPS).

The material phase composition of the AxB/HPY series catalysts was characterized by XRD using a Bruker D8 Advance diffractometer (Bruker AXS GmbH, Karlsruhe, Germany) operated at 40 kV and 40 mA, with a scanning rate of 2°/min over a 2θ range of 5–90°. Morphological features were examined by TEM on a Tecnai G2 F20 instrument (Thermo Fisher Scientific, Waltham, MA, USA); samples were ultrasonically dispersed in ethanol and deposited onto carbon-coated copper grids prior to imaging.

Textural properties, including specific surface area and pore size distribution, were determined by N_2_ physisorption at –196 °C using a Micromeritics TriStar II 2020 analyzer (Micromeritics Instrument Corporation, Norcross, GA, USA). Prior to measurement, samples were degassed at 350 °C under vacuum for 6 h. Surface area was calculated using the BET method, and pore size distribution was derived from the adsorption branch via the Barrett–Joyner–Halenda (BJH) model.

Acidic properties were evaluated by NH_3_-TPD and Py-FTIR. NH_3_-TPD was performed using a Tianjin Xianqua TP-5076 system (Tianjin Xianquan Instrument Co., Ltd., Tianjin, China); samples were pretreated at 600 °C under N_2_, saturated with NH_3_ at 100 °C, and then heated to 600 °C at 10 °C/min. Py-FTIR spectra were recorded on a Digilab spectrometer (Digilab LLC, Cambridge, MA, USA) after pyridine adsorption and subsequent desorption at 200 °C and 350 °C. Acid site densities were quantified based on integrated peak areas and molar extinction coefficients.

The chemical states of metal species in sulfided catalysts were analyzed by XPS. Binding energies were calibrated against the C 1s peak at 284.8 eV. Reducibility of oxide precursors was assessed by H_2_ temperature-programmed reduction (H_2_-TPR), with samples heated to 900 °C under a H_2_/Ar flow.

### 3.5. Catalytic Evaluation

Naphthalene hydrocracking was evaluated in a fixed-bed continuous-flow reactor. Catalyst particles (0.5 g, 40–60 mesh) were diluted with quartz sand (2.8 g). Reactions were conducted at 4.0 MPa total pressure, a weight hourly space velocity (WHSV) of 4 h^−1^ (based on naphthalene), and an H_2_/oil ratio of 600 (*v*/*v*). The feedstock was 5.0 wt% naphthalene dissolved in n-octane. Reaction temperatures ranged from 350 °C to 425 °C. Liquid products were collected after 4 h on stream for each temperature and analyzed using an Thermal TSQ 8000 EVO gas chromatography mass spectrometry (Thermo Fisher Scientific, Waltham, MA, USA) equipped with an HP-innowax capillary column (30 m × 0.32 mm × 0.25 μm). Carbon balances exceeded 95%. Naphthalene conversion (Conv.%) and product selectivities (Sel.%) were calculated as follows:

Conv.% = (1 − [Moles of Naphthalene in Product]/[Moles of Naphthalene in Feed]) × 100%

Sel.% = (Moles of Product i/Moles of All Detected Products) × 100%

BTX Yield.% = Conv.% × (Sel.Benzene + Sel.Toluene + Sel.Xylene)/100

## 4. Conclusions

Optimizing the spatial architecture of metal and acid sites within a hierarchical pore-structured catalyst is essential for selective naphthalene hydrocracking to BTX. The superior performance of Ni_1_W/HPY originates from a unique synergy: large WO_3_ crystallites located preferentially in mesopores and on external surfaces preserve microporosity, enabling efficient separation of hydrogenation and cracking functions. This structure enforces an optimal ‘hydrogenate-then-crack’ pathway, minimizing over-cracking and coke formation. While higher Ni loading improves W dispersion, it also induces Ni agglomeration and reduces sulfidation degree, lowering hydrogenation activity. Comparatively, Co_2_Mo/HPY exhibits higher Mo sulfidation but weaker acidity, resulting in inferior cracking performance. This study highlights the critical balance required between metal dispersion, sulfidation state, acid strength, and pore architecture in designing high-performance bifunctional catalysts for aromatic upgrading.

## Figures and Tables

**Table 1:** Pore Structure Parameters of A_x_B/HPY Series Catalysts.

Samples /HPY	Pore Size (nm)	V_total_ (cm^3^/g)	V_mic_ (cm^3^/g)	V_mes_ (cm^3^/g)	S_BET_ (m^2^/g)	S_mic_ (m^2^/g)	S_ext_ (m^2^/g)
HPY	7.5	0.45	0.26	0.19	709	554	155
Ni_2_Mo	5.8	0.27	0.07	0.20	258	170	88
Co_2_Mo	6.5	0.25	0.08	0.17	277	194	83
Ni_1_W	7.0	0.32	0.20	0.12	536	427	109
Ni_2_W	7.2	0.33	0.21	0.12	548	437	111
Ni_3_W	7.3	0.31	0.20	0.11	540	434	106
Ni_5_W	7.4	0.32	0.20	0.12	539	433	106

**Table 2:** Acid Properties of A_x_B/HPY Series Catalysts.

Samples /HPY	Total Acid μmol/g	Weak Acid, μmol/g			Strong Acid, μmol/g		
		L	B	L + B	L	B	L + B
Ni_2_Mo	619.2	72.0	158.1	230.1	165.7	223.4	389.1
Co_2_Mo	557.2	113.9	93.2	207.1	60.5	289.6	350.1
Ni_1_W	431.0	59.0	111.3	170.3	64.2	196.5	260.7
Ni_2_W	461.9	61.5	120.3	181.8	38.0	242.2	280.2
Ni_3.5_W	480.6	125.2	63.5	188.7	56.1	235.8	291.9
Ni_5_W	495.3	114.9	80.7	195.6	65.9	233.8	299.7

**Table 3:** Mo(W) XPS peak data of molybdenum (tungsten) species in sulfided A_x_B/HPY series catalysts.

Samples /HPY	Mo(W)^4+^%	Mo(W)^5+^%	Mo(W)^6+^%
Ni_2_Mo	48.8	7.1	44.1
Co_2_Mo	72.4	5.4	22.2
Ni_1_W	70.3	9.7	20.1
Ni_2_W	50.2	8.5	41.3
Ni_3.5_W	52.1	5.4	42.5
Ni_5_W	59.5	26.7	13.8

## Data Availability

The original contributions presented in this study are included in the article. Further inquiries can be directed to the corresponding authors.
